# Supplementary material for: DMRforPairs: identifying Differentially Methylated Regions between unique samples using array based methylation profiles
Source: BMC Bioinformatics. 2014 May 15;15:141. doi: 10.1186/1471-2105-15-141 (PMC4046028; doi:10.1186/1471-2105-15-141)
Supplement: Additional file 2 — DMRforPairs output for the comparison of A431-MCF7 and NA17018-NA17105. Please start from the HTML files in each folder. Available via the BMC Bioinformatics website. [file 1471-2105-15-141-S2.zip › 1394847754114233_MOESM2_ESM/A431_MCF7/figures/32168.pdf]

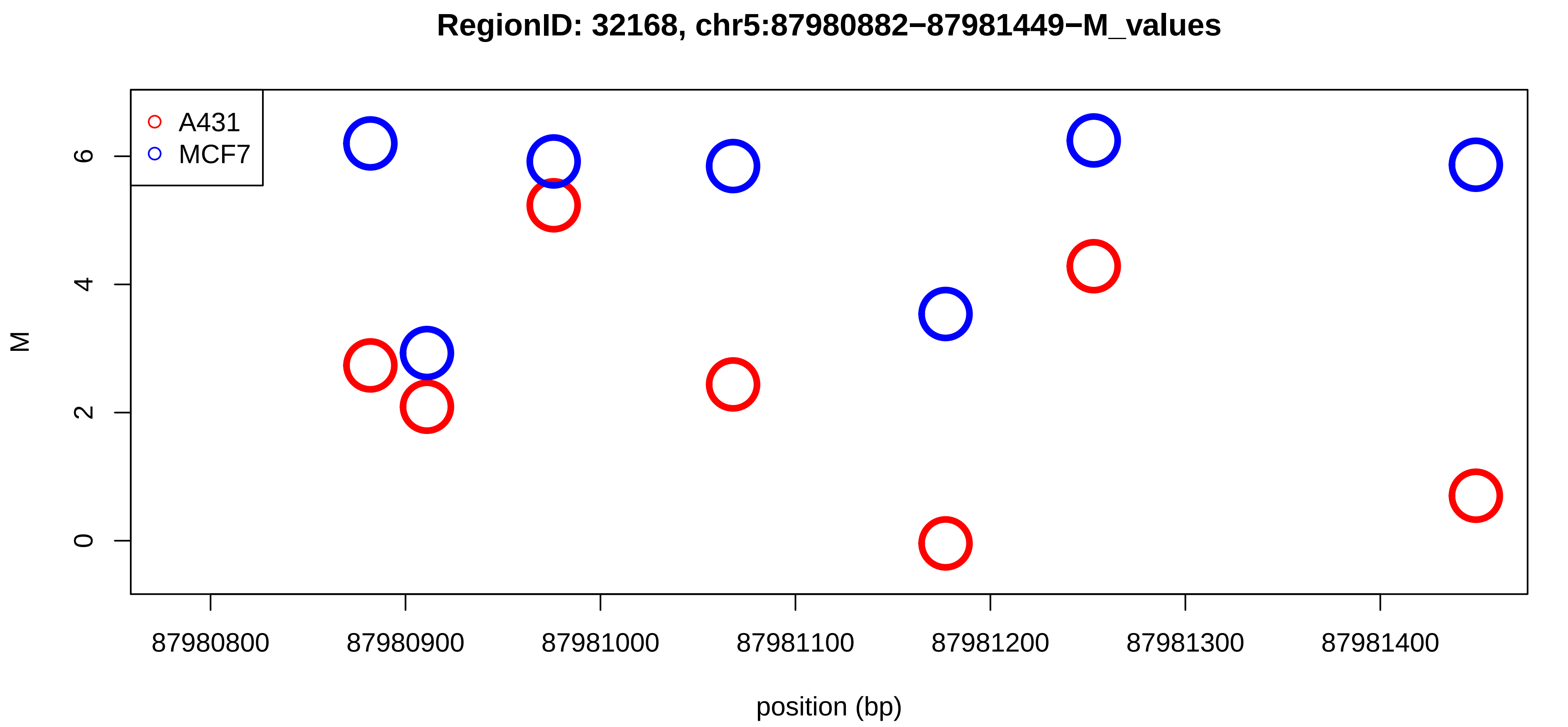
RegionID: 32168, chr5:87980882−87981449−M_values
A431
MCF7
M
0
2
4
6
87980800
87980900
87981000
87981100
87981200
87981300
87981400
position (bp)

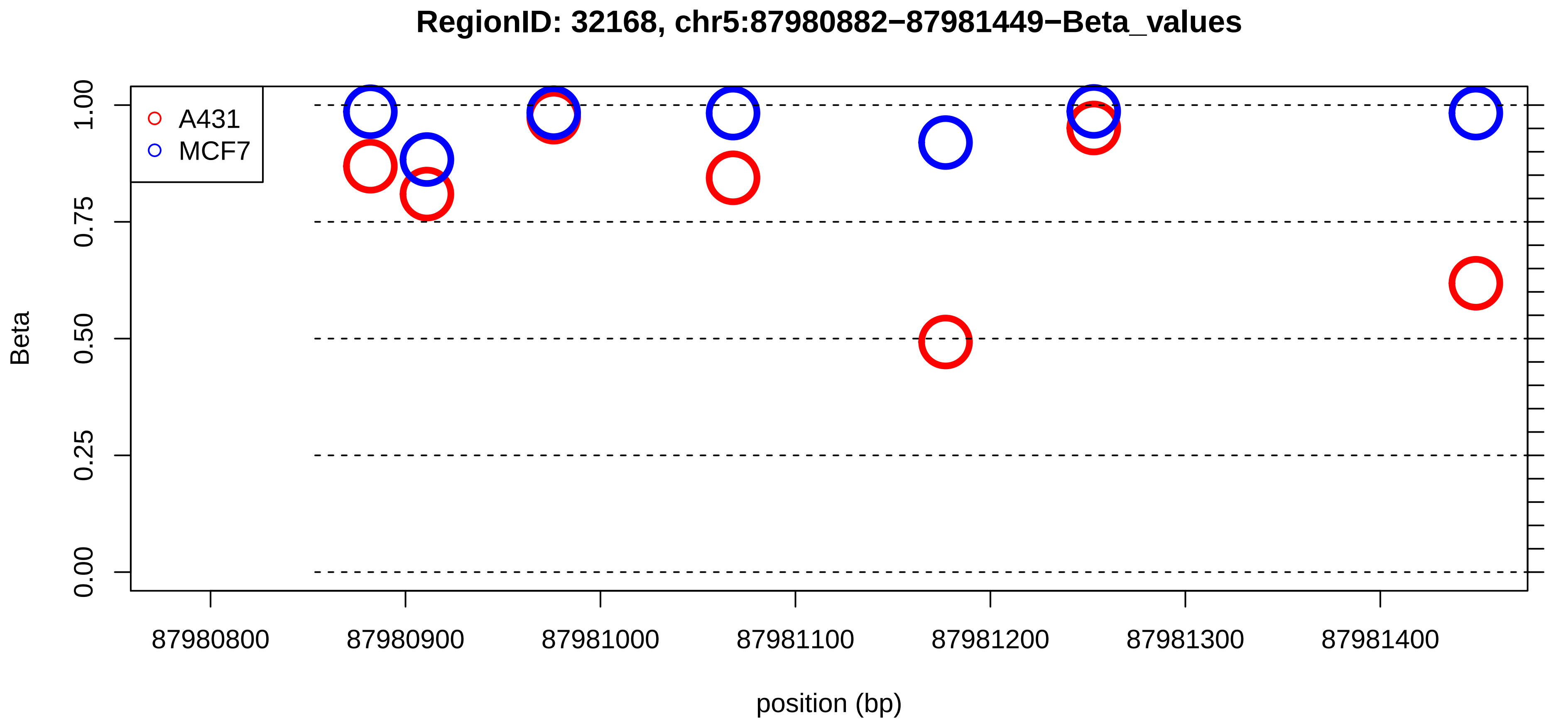
RegionID: 32168, chr5:87980882−87981449−Beta_values
A431
MCF7
Beta
0.00
0.25
0.50
0.75
1.00
87980800
87980900
87981000
87981100
87981200
87981300
87981400
position (bp)

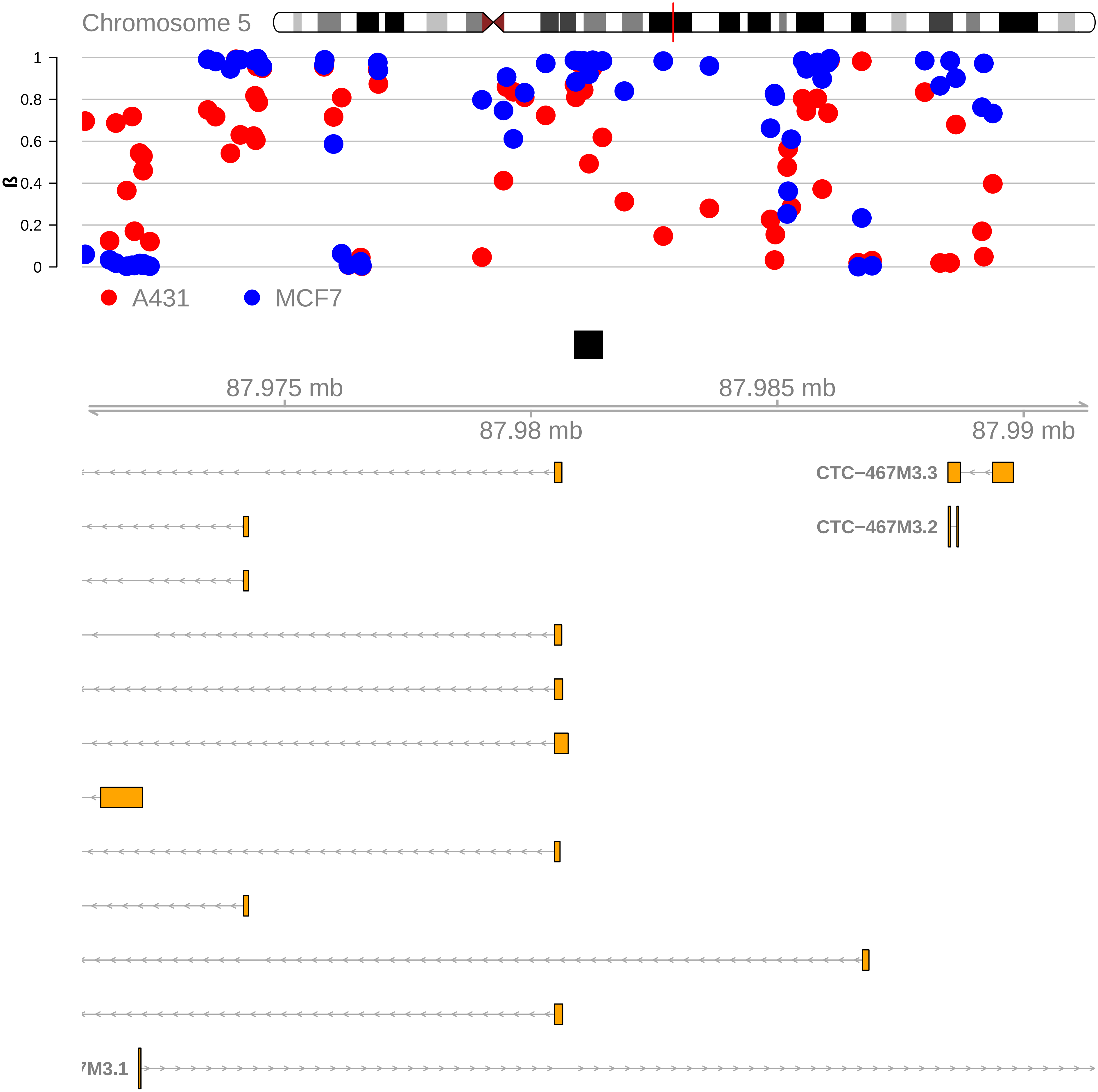
Chromosome 5
ß
1
0.8
0.6
0.4
0.2
0
A431
MCF7
87.975 mb
87.985 mb
87.98 mb
87.99 mb
CTC−467M3.3
CTC−467M3.2
7M3.1
